# Robber flies and hover flies (Insecta, Diptera, Asilidae and Syrphidae) in beech forests of the central Apennines: a contribution to the inventory of insect biodiversity in Italian State Nature Reserves

**DOI:** 10.3897/BDJ.11.e101327

**Published:** 2023-05-11

**Authors:** Alice Lenzi, Daniele Birtele, Silvia Gisondi, Mario Romano, Bruno Petriccione, Pierfilippo Cerretti, Alessandro Campanaro

**Affiliations:** 1 Consiglio per la ricerca in agricoltura e l’analisi dell’economia agraria – Centro di ricerca Difesa e Certificazione, Firenze, Italy Consiglio per la ricerca in agricoltura e l’analisi dell’economia agraria – Centro di ricerca Difesa e Certificazione Firenze Italy; 2 Dipartimento di Biologia e Biotecnologie ‘Charles Darwin’, Sapienza Università di Roma, Roma, Italy Dipartimento di Biologia e Biotecnologie ‘Charles Darwin’, Sapienza Università di Roma Roma Italy; 3 NBFC, National Biodiversity Future Center, Palermo, Italy NBFC, National Biodiversity Future Center Palermo Italy; 4 Carabinieri Biodiversità, Reparto di Verona – Centro Nazionale Carabinieri Biodiversità “Bosco Fontana", Marmirolo (Mantova), Italy Carabinieri Biodiversità, Reparto di Verona – Centro Nazionale Carabinieri Biodiversità “Bosco Fontana" Marmirolo (Mantova) Italy; 5 Raggruppamento Carabinieri Biodiversità, Reparto di Castel di Sangro, Castel di Sangro (L'Aquila), Italy Raggruppamento Carabinieri Biodiversità, Reparto di Castel di Sangro Castel di Sangro (L'Aquila) Italy; 6 Colonnello dei Carabinieri per la Biodiversità, nella riserva, Castel di Sangro, Italy Colonnello dei Carabinieri per la Biodiversità, nella riserva Castel di Sangro Italy

**Keywords:** insect diversity, biodiversity, Malaise trap, dataset, Zenodo repository, Diptera, robber flies, hover flies, sampling-event data.

## Abstract

**Background:**

The present paper describes a sampling-event dataset on species belonging to two families of Diptera (Syrphidae and Asilidae) collected between 2012 and 2019 in two Italian beech forests located in the central Apennines. The reference dataset consists of an annotated checklist and has been published on Zenodo. Syrphidae and Asilidae are two widespread and key ecological groups, including predator, pollinator and saproxylic species. Despite their pivotal role in both natural and man-made ecosystems, these families are still poorly known in terms of local distribution and open-access sampling-event data are rare in Italy.

**New information:**

This open-access dataset includes 2,295 specimens for a total of 21 Asilidae and 65 Syrphidae species. Information about the collection (e.g. place, date, methods applied, collector) and the identification (e.g. species name, author, taxon ID) of the species is provided. Given the current biodiversity crisis, the publication of checklists, sampling-event data and datasets on insect communities in open-access repositories is highly recommended, as it represents the opportunity to share biodiversity information amongst different stakeholders. Moreover, such data are also a valuable source of information for nature reserve managers responsible for monitoring the conservation status of protected and endangered species and habitats and for evaluating the effects of conservation actions over time.

## Introduction

Negative trends and remarkable changes in insect biodiversity have been recorded in the last decades (Wagner et al. 2021, Outhwaite et al. 2022). Recent analyses have also demonstrated that a steep decline is affecting both species richness and abundance (Hallmann et al. 2017, Habel et al. 2019, Powney et al. 2019, Hallmann et al. 2021). Thus, considering that the knowledge of natural communities is still superficial [e.g. more than 80% of species have not yet been described (Mora et al. 2011, Wilson 2017)] and given the unprecedented and pressing extinction rates (De Vos et al. 2014, Ceballos et al. 2015), an alarming scenario may result: populations or species could become extinct before we know their ecology, their distribution, their conservation status or even their existence (Costello et al. 2013, Raven and Wagner 2021).

In this context, a large amount of data is highly needed to achieve a sufficient awareness of species diversity, especially in species-rich insect groups (Wagner 2020). Unfortunately, standardised open-access datasets are not available for long-term analyses and spatial records of insects are rare (Rocha‐Ortega et al. 2021). Furthermore, the already published datasets are rarely fully accessible or reusable (Boeckhout et al. 2018) and do not comply with Open Science and FAIR Data policies (Wilkinson et al. 2016). In particular, in the Italian research scenario, sharing raw data is not really a common practice. For example, sampling-event data or checklists are often published as appendices in non-open access journals, published in local newsletters and gazetteers or not even considered worthy of publication (e.g. data collected through project actions). Dealing with nature conservation in protected areas, a communication gap lies between researchers and site managers, who often host sampling activities carried out for scientific purposes without receiving any feedback (McNie 2007, Gibbons et al. 2008, Merkle et al. 2019). Instead, occurrence data from the above-mentioned activities could be extremely valuable in order to build up a solid knowledge on biodiversity and community structure of protected areas, both of which are the basis for monitoring of the conservation status of species, evaluating the impact of conservation actions and planning appropriate management strategies. In addition, the extrapolation of occurrence data on protected species represents baseline information for the designation of Natura 2000 sites and the national reporting under the Habitats Directive (92/43/EEC). This given, it is therefore important to set up long-term studies and develop open and shared historical datasets that will allow to know and to monitor the biodiversity composition within a given area (Mirtl and Krauze 2007, Campanaro and Parisi 2021, Minelli et al. 2021). However, recent projects and initiatives aiming at the assessment of insect diversity have been launched, both at national level (e.g. Hausmann et al. (2020), Karlsson et al. (2020), Birtele (2021), Sommaggio and Birtele (2021), Bologna et al. (2022)) or with a focus on specific functional groups (e.g. pollinators, Potts et al. (2015), Potts et al. (2020)).

In the above scenario, Diptera constitutes an extremely challenging taxon. In fact, it is one of the largest insect orders on Earth, with hundreds of thousands of undescribed species in addition to the approximately 160,000 currently named ones (Courtney et al. 2017, Wiegmann and Yeates 2017) belonging to approx. 180 families worldwide (Bertone et al. 2008, Brown 2009, Courtney and Cranston 2015). Flies exhibit an impressive diversity of biological traits, such as feeding habits, behaviour and life histories, due to their ability to exploit several important ecological niches (i.e. they include scavengers, predators, pollinators, parasites and parasitoids). However, as proved by recent studies, this species diversity is still greatly underestimated (Hebert et al. 2016, Forbes et al. 2018), as well as the bionomy, distribution (especially at local scale) and conservation status of most taxa. Furthermore, Diptera are often excluded from studies, checklists and assessments on local biodiversity, mainly due to the difficulties in their identification process, which is usually time-consuming and requires highly skilled taxonomists. Asilidae and Syrphidae are two of the most species-rich dipteran families (both including approximately 7,000 known species) (Pape et al. 2011) with a worldwide distribution. They include pollinators, predators and saproxylic species inhabiting both natural and man-made environments (Dunn et al. 2020, Veríssimo et al. 2020). Asilidae (‘robber flies’ or ‘assassin flies’) are predators both during the larval stages and as adults (Musso 1983, Dennis et al. 2013), a rare feature amongst Diptera. Thus, they are key species directly controlling insect populations and maintaining community equilibrium (Wei et al. 1995). Syrphidae (‘hover flies’ or ‘flower flies’) are known to be one of the most important dipteran pollinator groups (Larson et al. 2012), as the adults are mainly anthophilous (Sack 1932, Vockeroth and Thompson 1987). They can be considered good environmental indicators in Europe (Sommaggio 1999, Maleque et al. 2009); in fact, the taxonomy, as well as the ecological characteristics of most species, are well known (Speight 2014). In addition, the larval stages of some groups are saproxylic, thus closely associated with veteran trees and dead wood (Fayt et al. 2006, Ricarte et al. 2009, Birtele and Hardersen 2012). Other groups are instead predators in the immature stages, mainly as aphid eaters and are potential biological control agents (Schneider 1969, Bugg et al. 2008, Dunn et al. 2020).

The present paper describes a dataset of Asilidae and Syrphidae species occurrences in field collections carried out between 2012 and 2019 in two beech forest areas in central Italy, included in the protected areas “Foresta Demaniale Regionale Chiarano-Sparvera” and "Vallone di Teve" in the State Nature Reserve “Monte Velino" (L’Aquila Province, Abruzzo).

## General description

### Purpose

Our overall purpose is to promote the collection and publication of raw data and information on insect communities inhabiting the Italian State Nature Reserves. In the present publication, we describe a dataset on sampling-event data of species belonging to two Diptera families. This dataset could be considered as a starting point for the implementation of additional future sampling campaigns in order to establish long-term data series for biodiversity surveillance and to obtain a reliable source of information for the management and conservation of the natural environment.

## Project description

### Title

Specimens were collected within three projects: (i) LIFE09 ENV/IT/000078 - Management of Forests, Carbon and Biodiversity (ManFor C.BD), (ii) LIFE17 ESC/IT/001 360 - Volunteers for monitoring forest biodiversity in the Italian Natura 2000 Network (LIFE ESC360) and (iii) a collaboration agreement between Sapienza Università di Roma and the Carabinieri special division “Laboratorio Nazionale Tassonomia e Bioindicazione Invertebrati - Reparto Biodiversità Carabinieri di Verona (LanaBit)”.

### Personnel

General information about the above-mentioned projects and the involved institutions is provided in Table 1.

## Sampling methods

### Sampling description

Samplings were performed using Malaise traps and hand collections (i.e. net collections). A total of 17 traps were installed and activated (Table 2) and the solution employed for preserving the specimens was 70% ethanol. Traps were emptied every 15 days by the project staff or volunteers (i.e. during LIFE ESC360).

### Step description

The collected specimens were analysed in well-equipped laboratories: they were preliminarily sorted at family level to select specimens belonging to the target groups, then identified to species level.

## Geographic coverage

### Description

**Figure 1.**
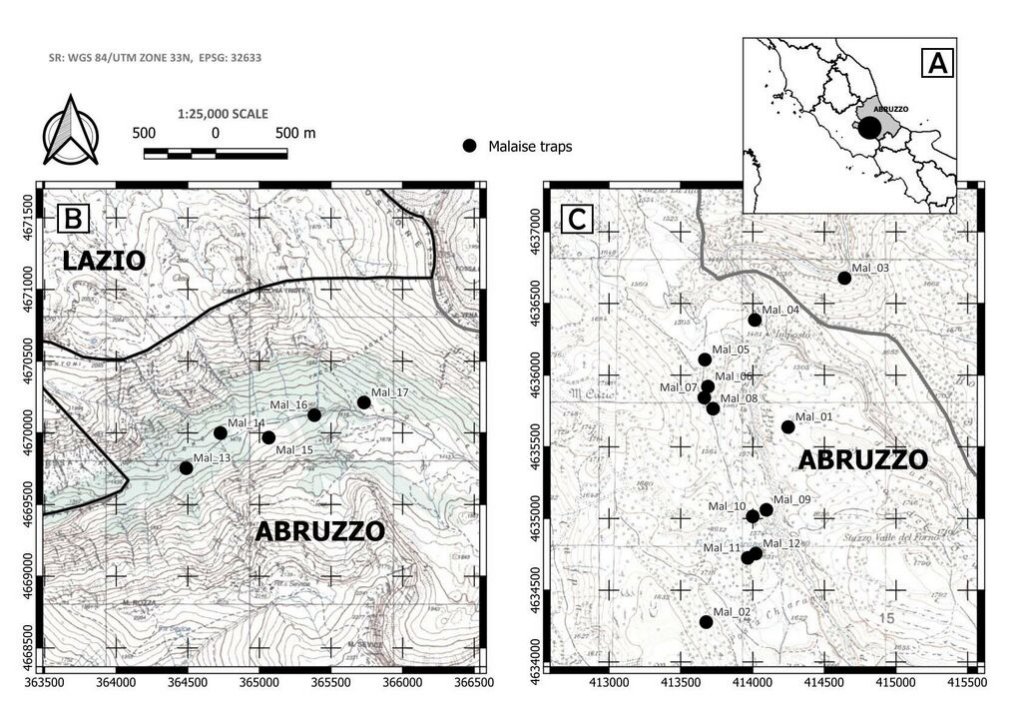
Study areas (scale bar and north pointer are reported for figures B and C). **A.** Location of the study area in Italy. **B.** Study area “Vallone di Teve”, black dots correspond to the five Malaise traps of the area. **C.** Study area “F.D. Chiarano-Sparvera”, black dots correspond to the 12 Malaise traps of the area. The coordinates of the traps are reported in Table 2.

The dataset includes information on species collected in two beech forests of the central Apennines, in the Abruzzo Region (Italy) (Fig. 1). Forest typologies belong to the EUNIS habitat type *Fagus* forest on non-acid soils (European Environmental Agency, EUNIS Habitat Classification 2021) and to the priority habitat 9210* (*Apennine beech forests with *Taxus* and *Ilex*, EU Habitats Directive 92/43/EEC). The forests are included in two protected areas managed by Arma dei Carabinieri, Comando Unità Forestali, Ambientali e Agroalimentari (CUFA). The first area is the Foresta Demaniale Regionale Chiarano-Sparvera (L'Aquila Province) (from now F.D. Chiarano-Sparvera), partially overlapping the Special Area of Conservation (SAC) IT7110205 “Parco Nazionale d'Abruzzo”. The second area is Vallone di Teve (L'Aquila Province), a glacial mountain valley included in the protected area State Nature Reserve “Monte Velino”, the SAC IT7110206 “Monte Sirente e Monte Velino” and the Special Protection Area (SPA) IT7110130 “Sirente Velino”.

The samplings were carried out in clearings within the forests, between 1,397 m a.s.l. and 1,746 m a.s.l.

### Coordinates

41.8598 and 42.1725 Latitude; 13.3594 and 13.9713 Longitude.

## Taxonomic coverage

### Description

The published dataset contains records of individuals belonging to Syrphidae and Asilidae (Diptera) inhabiting beech forests. Each collected specimen was identified to species level by an expert taxonomist (DB), though only the genus name is given in case of uncertain species identification.

## Temporal coverage

**Data range:** 2012-5-23 – 2012-8-17; 2014-5-14 – 2014-10-06; 2019-7-05 – 2019-10-14.

### Notes

Samplings were carried out in the following periods: from May to August 2012, from May to October 2014 and from July to October 2019.

## Collection data

### Specimen preservation method

Specimens were preserved in ethanol or mounted on cards or pinned and dried. The entomological material is currently deposited at “Laboratorio Nazionale Tassonomia e Bioindicazione Invertebrati - Reparto Biodiversità Carabinieri di Verona” (LanaBit) (Verona, Italy).

## Usage licence

### Usage licence

Creative Commons Public Domain Waiver (CC-Zero)

## Data resources

### Data package title

Asilidae and Syrphidae (Insecta: Diptera) inhabiting beech forests in central Italy.

### Number of data sets

1

### Data set 1.

#### Data set name

Asilidae and Syrphidae (Insecta: Diptera) inhabiting beech forests in central Italy.

#### Data format

The dataset is available as .csv file.

#### Download URL

https://doi.org/10.5281/zenodo.7593442

#### Description

The dataset “Asilidae and Syrphidae (Insecta: Diptera) inhabiting beech forests in central Italy” was published on Zenodo (https://doi.org/10.5281/zenodo.7593442) as an open access file and under the Creative Commons Attribution 4.0 International Licence. The file consists of annotated checklists of robber flies (Asilidae) and hover flies (Syrphidae) (Insecta, Diptera).

The terms used for naming the fields in the dataset follow the Darwin Core standard (Darwin Core Maintenance Group 2021: Wieczorek et al. (2012) (https://dwc.tdwg.org/terms/). The harmonisation of the dataset concerning information about taxa identification, authorship, LSID (i.e. an identifier for the nomenclatural details of the scientific names) and the massive upgrading of the related identifiers in Zenodo record were performed using the dplyr taxize and zen4r packages in R, respectively (Chamberlain and Szöcs 2013, Blondel and Barde 2020).

The list of terms used in the present dataset are briefly described below: catalogNumber (i.e. a unique identifier of the record), order, family, genus, epithet, scientificName (genus species or genus of the biological entity), verbatimIdentification, scientificNameAuthorship, individualCount (total number of the individuals sampled), sex, disposition (where the samples are located at the edge), year, month (in which the sample was collected), habitat (habitat type according to EUNIS habitat classification 2021), samplingProtocol, Country, decimalLatitude, decimalLongitude, geodeticDatum, locality, minimumElevationInMetres, maximumElevationInMetres, recordedBy, identifiedBy, scientificNameID (i.e. the unique identifier for the species; if the specific names do not have a match in the Fauna Europea Database, the field is blank and the “scientificName” reported corresponds to the name indicated by the expert entomologist), taxonID (i.e. the identifier for the set of taxon information), nameAccordingTo (i.e. the reference to the source in which the specific taxon concept circumscription is defined or implied).

The dataset contains 1,031 records for a total of 2,295 specimens (407 asilids and 1,888 syrphids), corresponding to 86 known species (21 asilids and 65 syrphids) belonging to 41 genera, plus 19 syrphid taxa only identified at genus level. As reported in Tables 1 and 2, samplings were carried out in different periods and with a different sampling effort each year; thus, it is not possible to compare the obtained results. However, in Table 3, the results obtained in the two study areas and for the three years of samplings are summarised with the indication of the percentage of unique taxa (i.e. species found only in a specific year/area).

**Data set 1.** 

Column label	Column description
catalogNumber	An identifier of the occurrence within the dataset.
order	The full scientific name of the order in which the taxon is classified.
family	The full scientific name of the family in which the taxon is classified.
genus	The full scientific name of the genus in which the taxon is classified.
specificEpithet	The name of the epithet in the scientificName (e.g. bombylans for scientificName "Volucella bombylans").
scientificName	The full scientific name of the taxon.
verbatimIdentification	A string representing the taxonomic identification as it appeared in the original record.
taxonRank	The lower taxonomic rank assigned to the identified specimen (e.g. subspecies, species, genus, tribe).
scientificNameAuthorship	The authorship information for the scientificName.
basisOfRecord	The specific nature of the data record (e.g. preserved specimens, fossil specimen, living specimen, occurrence, observed event).
individualCount	The number of individuals of the same species collected in the same trap at the same time.
sex	The sex of the collected specimen(s).
disposition	The current state of the specimen(s).
eventDate	The date interval during which the specimen(s) was collected.
habitat	The EUNIS category of the habitat in which the specimen(s) was collected.
samplingProtocol	The names of the methods or protocols used during the sampling.
country	The name of the country in which the specimen(s) was collected.
decimalLatitude	The geographic latitude (in decimal degrees, EPSG:4326 - WGS84) of the geographic centre in which the specimen(s) was collected.
decimalLongitude	The geographic latitude (in decimal degrees, EPSG:4326 - WGS84) of the geographic centre in which the specimen(s) was collected.
geodeticDatum	The ellipsoid, geodetic datum or spatial reference system (SRS), upon which the geographic coordinates given in decimalLatitude and decimalLongitude are based.
coordinateUncertaintyInMetres	The horizontal distance (in metres) from the given decimalLatitude and decimalLongitude describing the smallest circle.
locality	The specific description of the place in which the sampling was carried out.
minimumElevationInMetres	The lower limit of the range of elevation (above sea level), in metres.
maximumElevationInMetres	The higher limit of the range of elevation (above sea level), in metres.
recordedBy	The person or the group responsible for collecting the specimen(s).
identifiedBy	Person who assigned the Taxon to the collected specimen(s).
institutionCode	The name (or acronym) in use by the institution having custody of the specimen(s).
scientificNameID	The identifier for the nomenclatural (not taxonomic) details of a scientific name.
taxonID	The global unique identifier for the set of taxon information (data associated with the Taxon class).
nameAccordingTo	The identifier for the source in which the specific taxon concept circumscription is defined or implied.

## Figures and Tables

**Table 1.** List of the projects under which samplings were carried out. Project name, duration and partners names, as well as collection periods and names of the study areas are reported.

**Project name**	**Project duration**	**Partners**	**Sampling period**	**Study area**
LIFE09 ENV/IT/000078 ‘LIFE ManFor C.BD’	2010 – 2015	· CNR – National Research Council · CREA – Council for Agricultural Research and Economics · Molise Region · Slovenian Forestry Institute · University of Molise, Department of Science and Technology for Environment and Territory · Veneto Region	May – Aug 2012 May – Sept 2014	F.D. Chiarano-Sparvera
LIFE17 ESC/IT/001 ‘LIFE ESC360’	2018 – 2022	· Comando Unità Forestali, Ambientali e Agroalimentari Carabinieri (CUFA) · D.R.E.Am. – Italia Soc. Coop. Agr. · CREA - Council for Agricultural Research and Economics	July – Oct 2019	Vallone di Teve
Scientific activities carried out under collaboration agreement	2019 – 2020	· Sapienza Università di Roma, ‘Charles Darwin’ Department of biology and biotechnologies · Laboratorio Nazionale Tassonomia e Bioindicazione Invertebrati - Ufficio Reparto Biodiversità Carabinieri di Verona (LanaBit)	July – Oct 2019	F.D. Chiarano-Sparvera

**Table 2.** Information on Malaise traps. Trap ID, name of the study area in which the trap was installed, coordinates in EPSG:4326 - WGS84 (DD.DDDD°), altitude and the period of sampling are reported.

**Trap ID**	**Study area**	**Geographical Coordinates**		**Altitude (m)**	**Sampling period**
		**Latitude**	**Longitude**		
Mal_01	F. D. Chiarano-Sparvera	41.8680	13.9667	1,613	May – Aug 2012 May – Sept 2014
Mal_02	F. D. Chiarano-Sparvera	41.8557	13.9600	1,746	June – Aug 2012 June – Oct 2014
Mal_03	F. D. Chiarano-Sparvera	41.8774	13.9713	1,397	July – Oct .2019
Mal_04	F. D. Chiarano-Sparvera	41.8747	13.9638	1,490	Juy – Oct 2019
Mal_05	F. D. Chiarano-Sparvera	41.8722	13.9596	1,542	July – Oct 2019
Mal_06	F. D. Chiarano-Sparvera	41.8705	13.9599	1,552	July – Oct 2019
Mal_07	F. D. Chiarano-Sparvera	41.8698	13.9596	1,566	July – Oct 2019
Mal_08	F. D. Chiarano-Sparvera	41.8691	13.96038	1,553	July – Oct 2019
Mal_09	F. D. Chiarano-Sparvera	41.8628	13.9650	1,587	July – Oct 2019
Mal_10	F. D. Chiarano-Sparvera	41.8624	13.9638	1,611	July – Oct 2019
Mal_11	F. D. Chiarano-Sparvera	41.8598	13.9634	1,639	July – Oct 2019
Mal_12	F. D. Chiarano-Sparvera	41.8601	13.9641	1,632	July – Oct 2019
Mal_13	Vallone di Teve	42.1682	13.3595	1,453	July – Oct 2019
Mal_14	Vallone di Teve	42.1704	13.3623	1,479	July – Oct 2019
Mal_15	Vallone di Teve	42.1702	13.3664	1,513	July – Oct 2019
Mal_16	Vallone di Teve	42.1717	13.3702	1,548	July – Oct 2019
Mal_17	Vallone di Teve	42.1725	13.3744	1,637	July – Oct 2019

**Table 3.** Detailed information on the number of specimens and species collected per year and study area.

**Year**	**Area**	**N of Malaise traps**	**Number of species**		**N of specimens**	**N of Asilidae species**	**N of Asilidae specimens**	**N of Syrphidae species**	**N of Syrphidae specimens**
			**Total**	**Unique species**					
2012	F. D. Chiarano-Sparvera	2	21	10%	87	7	30	14	57
2014	F. D. Chiarano-Sparvera	2	40	40%	584	11	73	39	511
2019	F. D. Chiarano-Sparvera	10	46	52%	1,224	14	285	32	939
2019	Vallone di Teve	5	47	51%	400	4	19	43	381
